# Supplementary figures and images for: Regulatory Programmes Driving Suberin Plasticity Under Aluminium Stress in Barley Roots
Source: Plant Cell Environ. 2025 Jul 17;48(11):7775–91. doi: 10.1111/pce.70075 (PMC12502035; doi:10.1111/pce.70075)

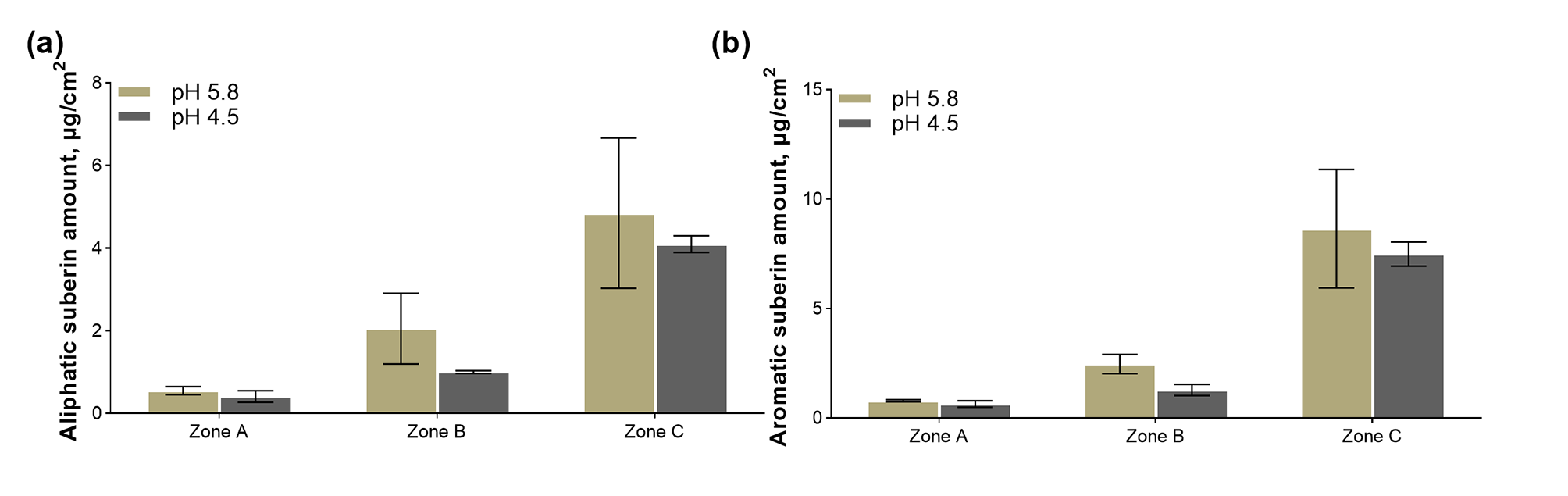

Supplement: Supplementary file 1 — FS1. [file PCE-48-7775-s001.tif]

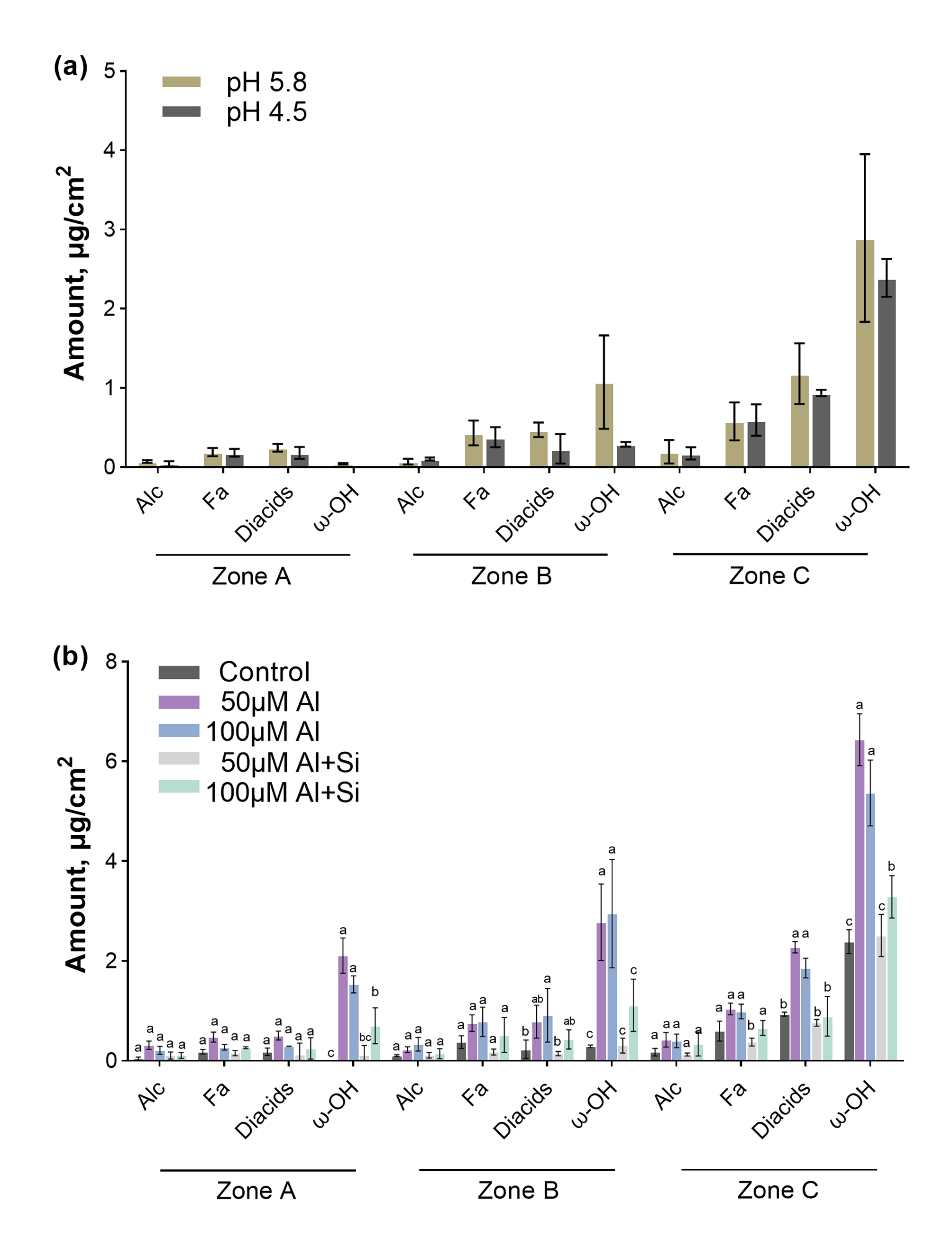

Supplement: Supplementary file 2 — FS2. [file PCE-48-7775-s005.tif]

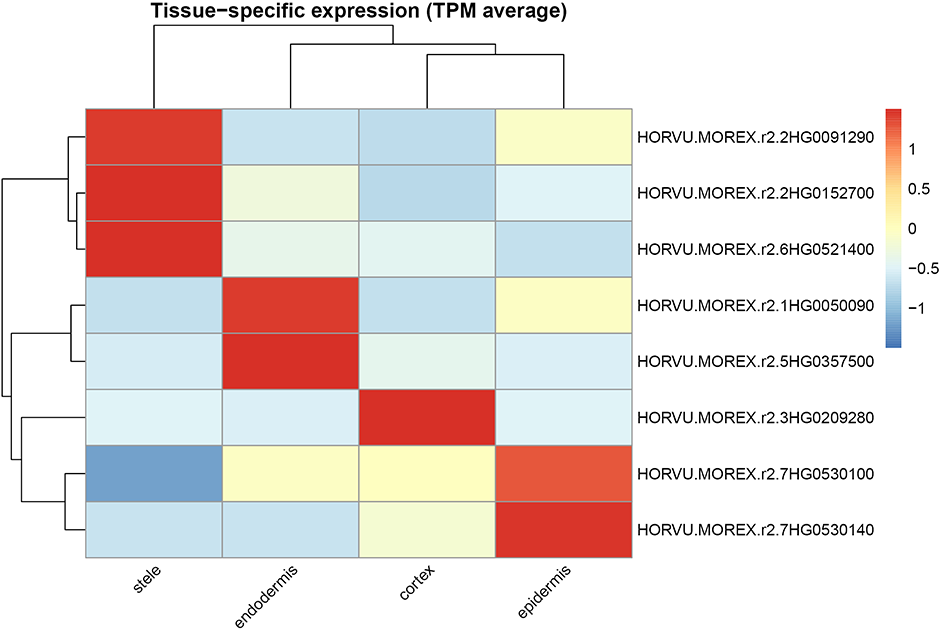

Supplement: Supplementary file 3 — FS3. [file PCE-48-7775-s003.tif]

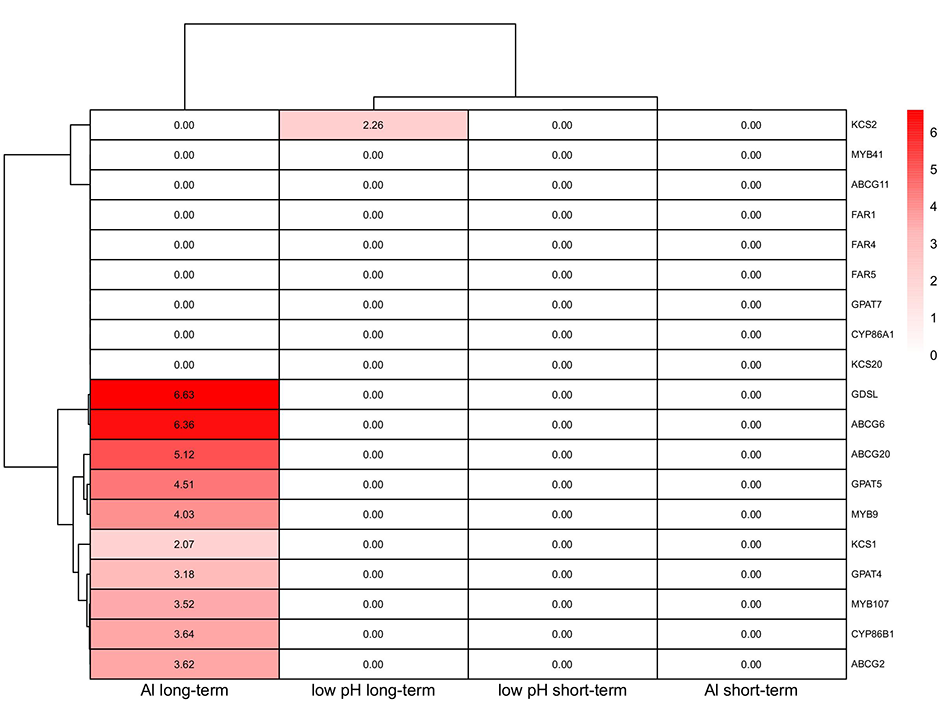

Supplement: Supplementary file 4 — FS4. [file PCE-48-7775-s010.tif]

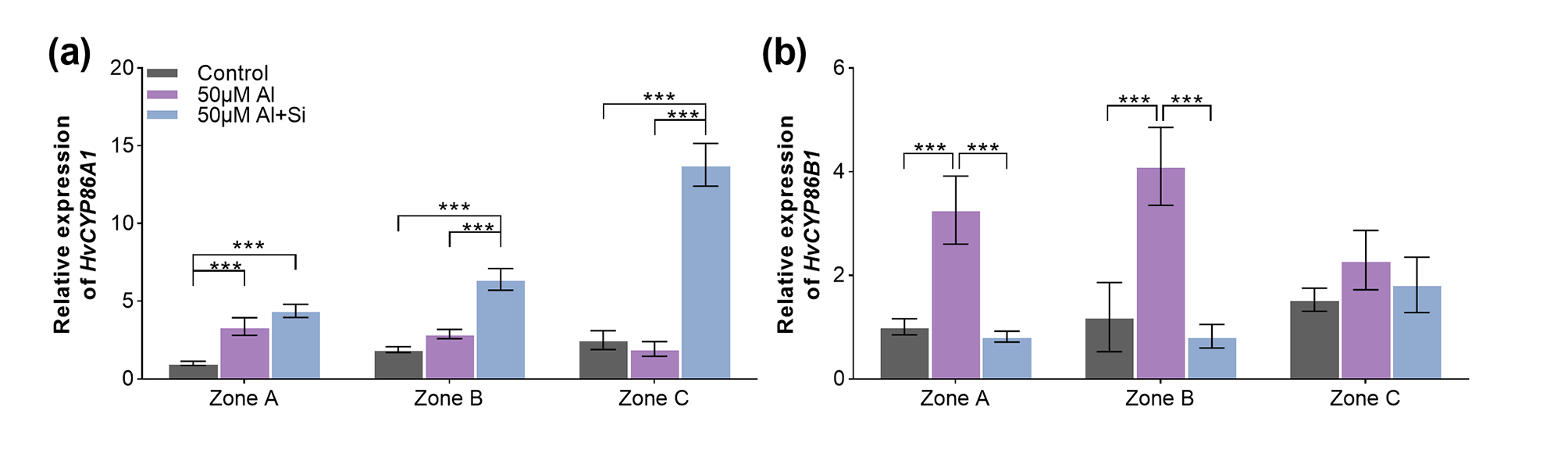

Supplement: Supplementary file 5 — FS5. [file PCE-48-7775-s011.tif]

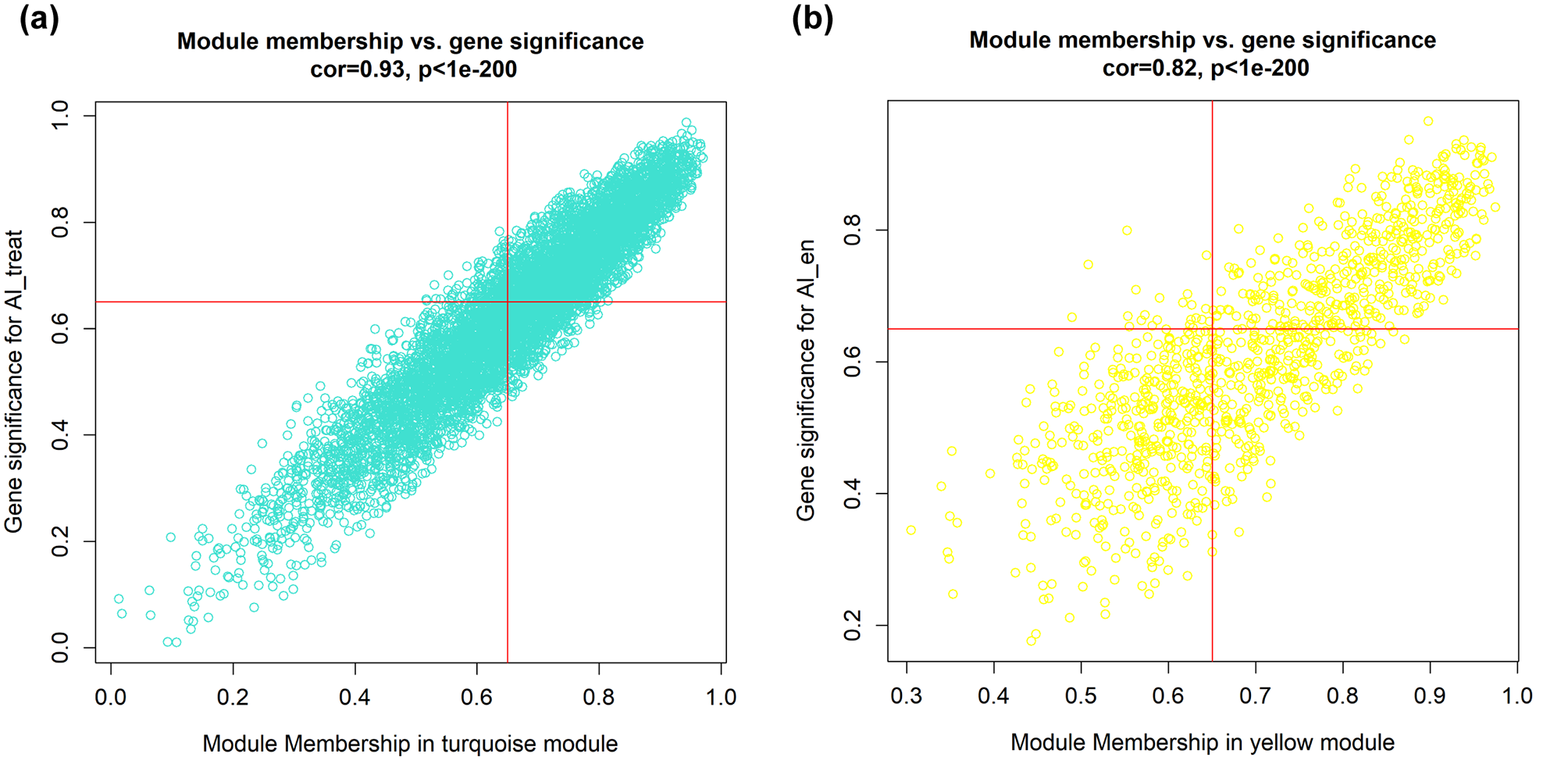

Supplement: Supplementary file 6 — FS6. [file PCE-48-7775-s016.tif]

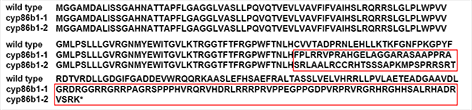

Supplement: Supplementary file 7 — FS7. [file PCE-48-7775-s019.tif]

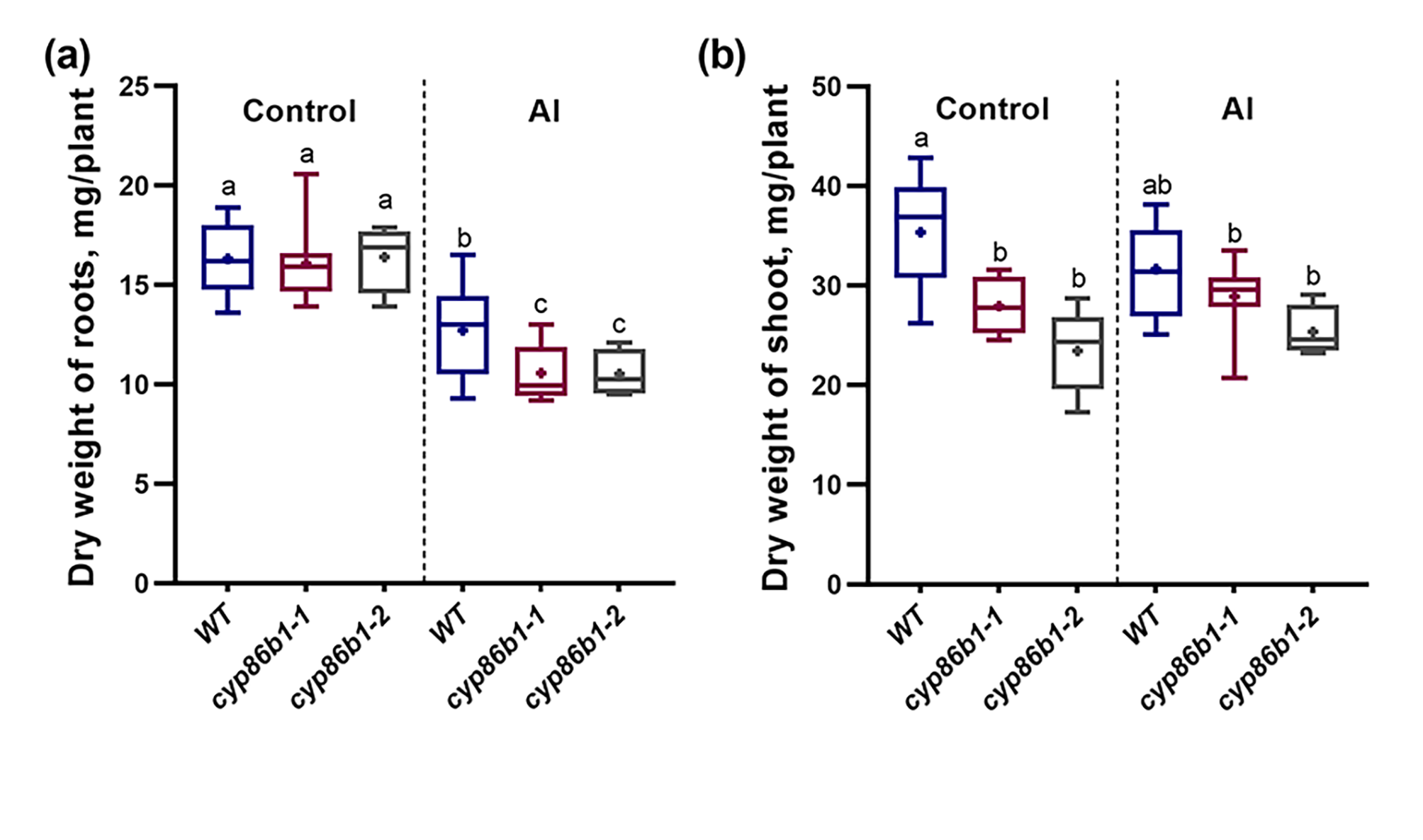

Supplement: Supplementary file 8 — FS8. [file PCE-48-7775-s012.tif]

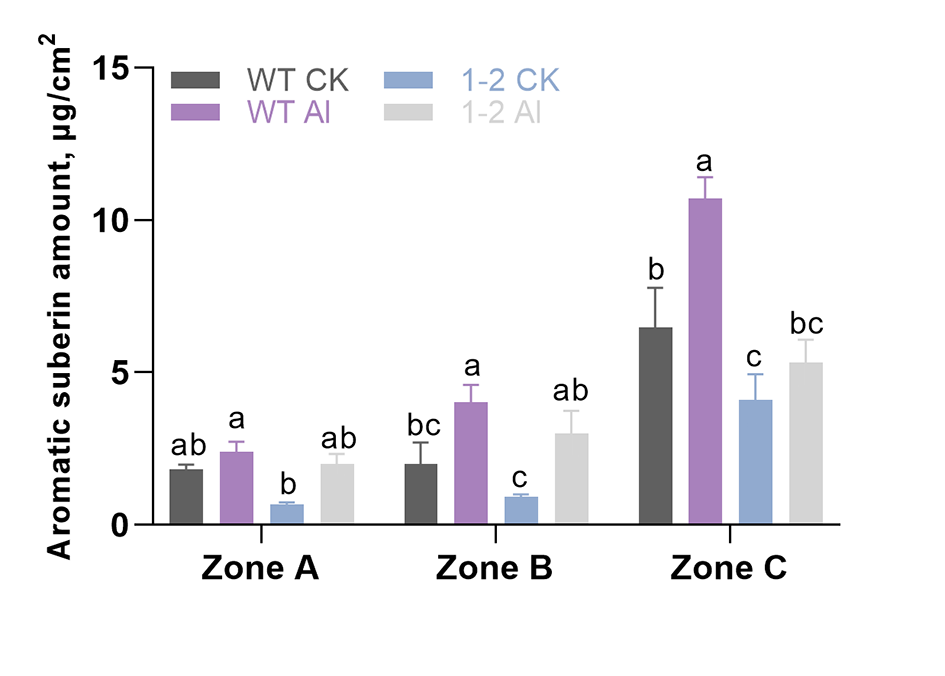

Supplement: Supplementary file 9 — FS9. [file PCE-48-7775-s009.tif]

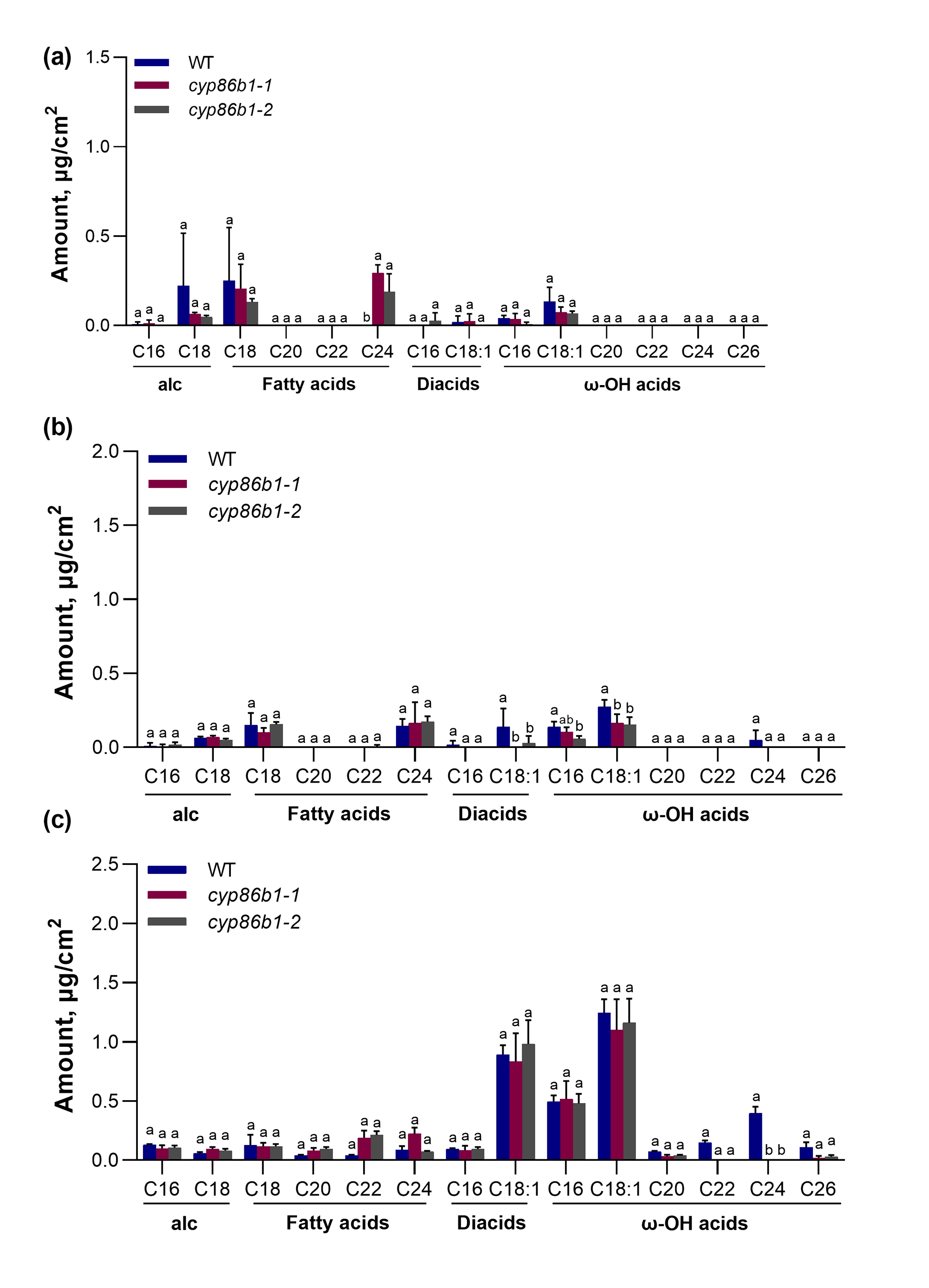

Supplement: Supplementary file 10 — FS10. [file PCE-48-7775-s021.tif]

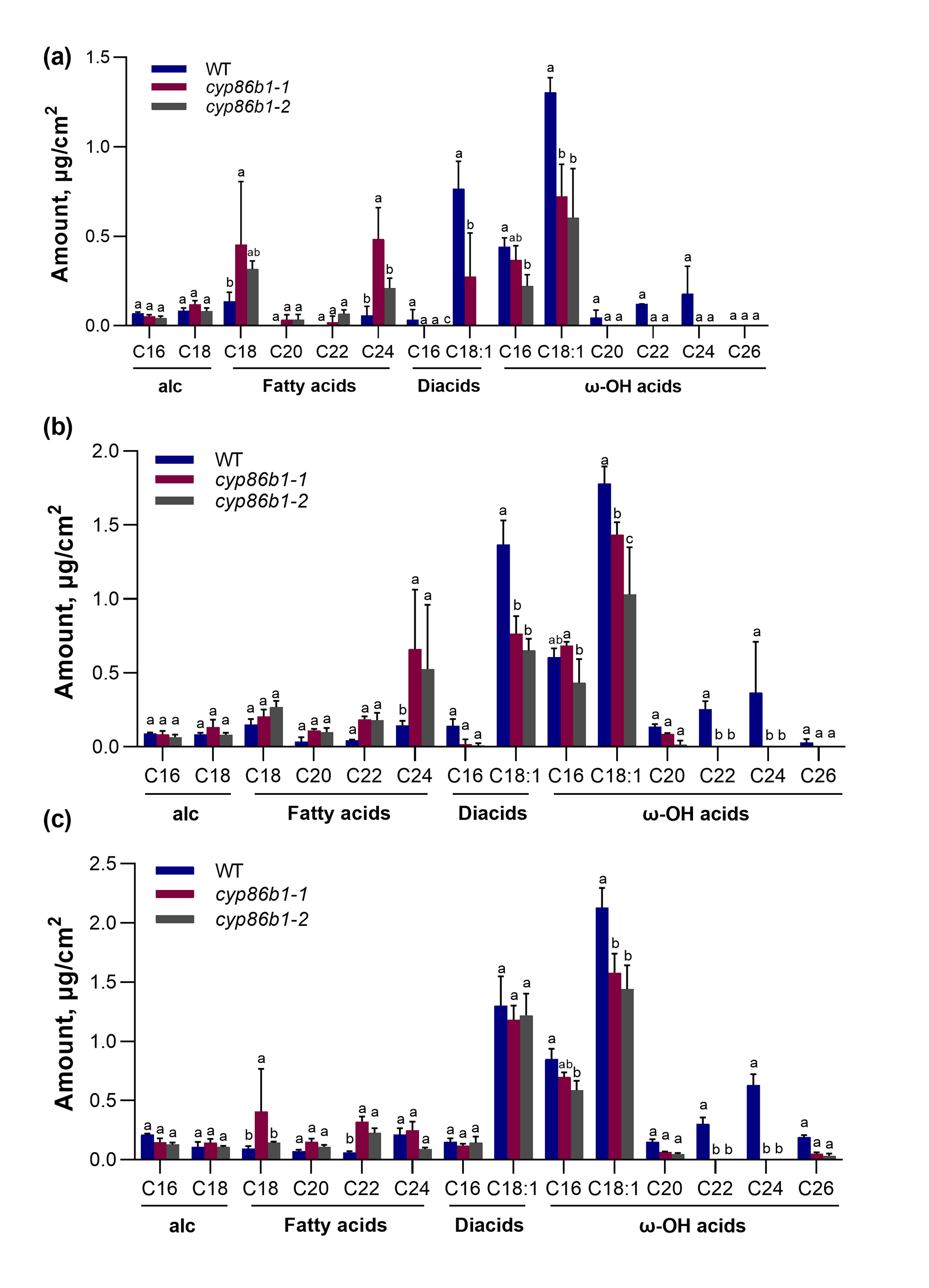

Supplement: Supplementary file 11 — FS11. [file PCE-48-7775-s014.tif]

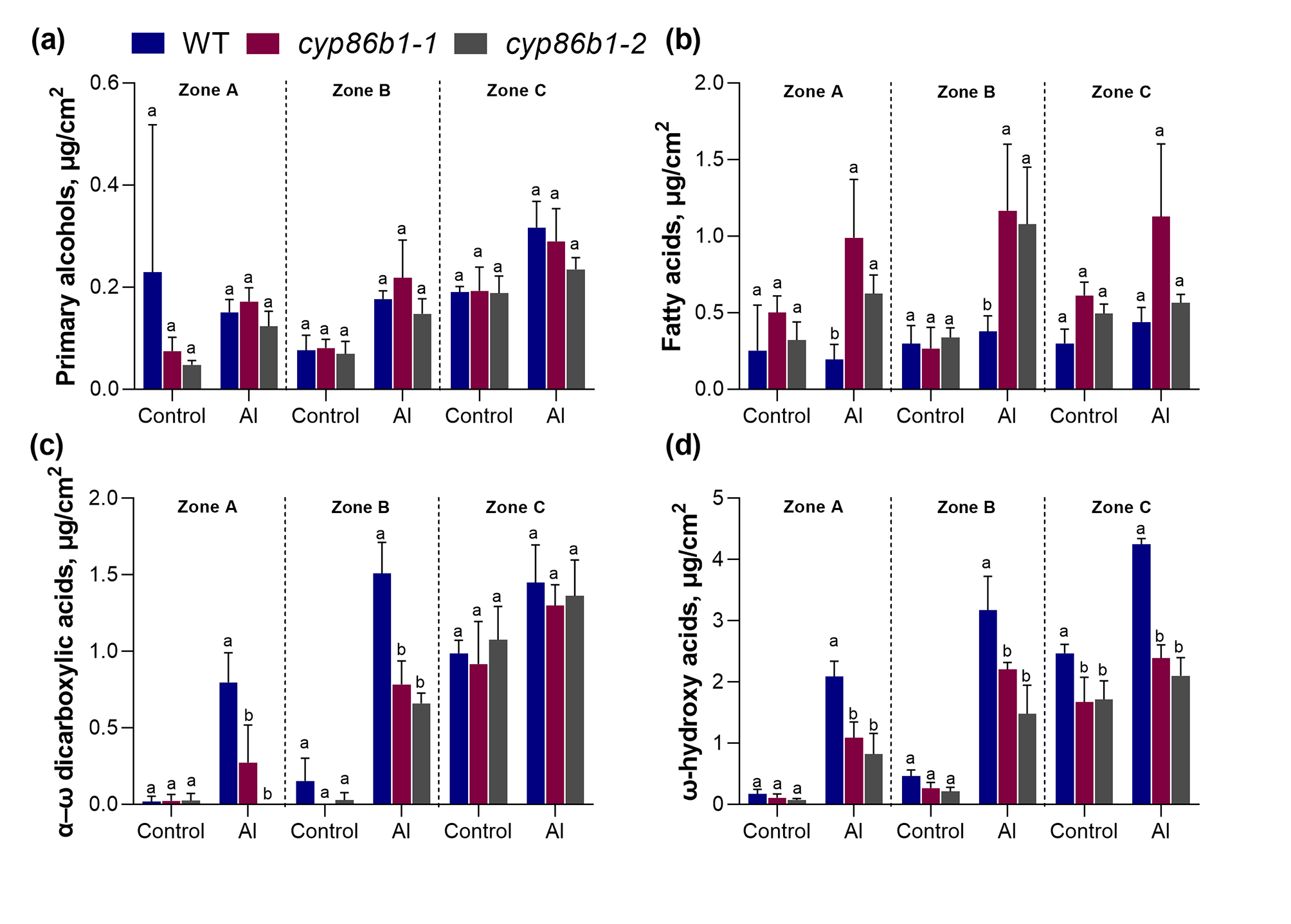

Supplement: Supplementary file 12 — FS12. [file PCE-48-7775-s004.tif]

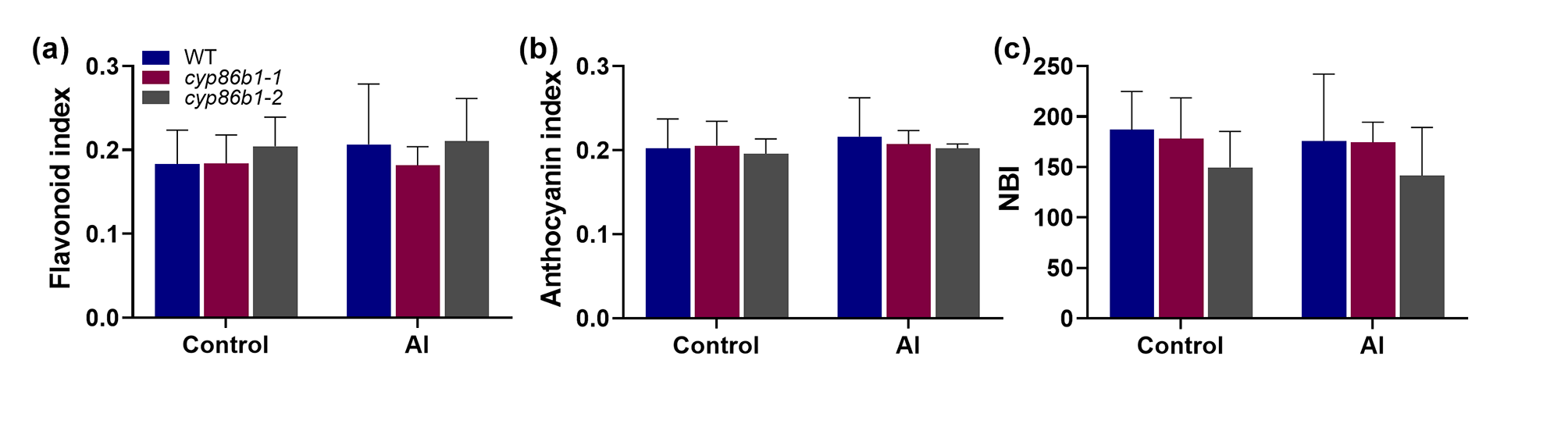

Supplement: Supplementary file 13 — FS13. [file PCE-48-7775-s013.tif]

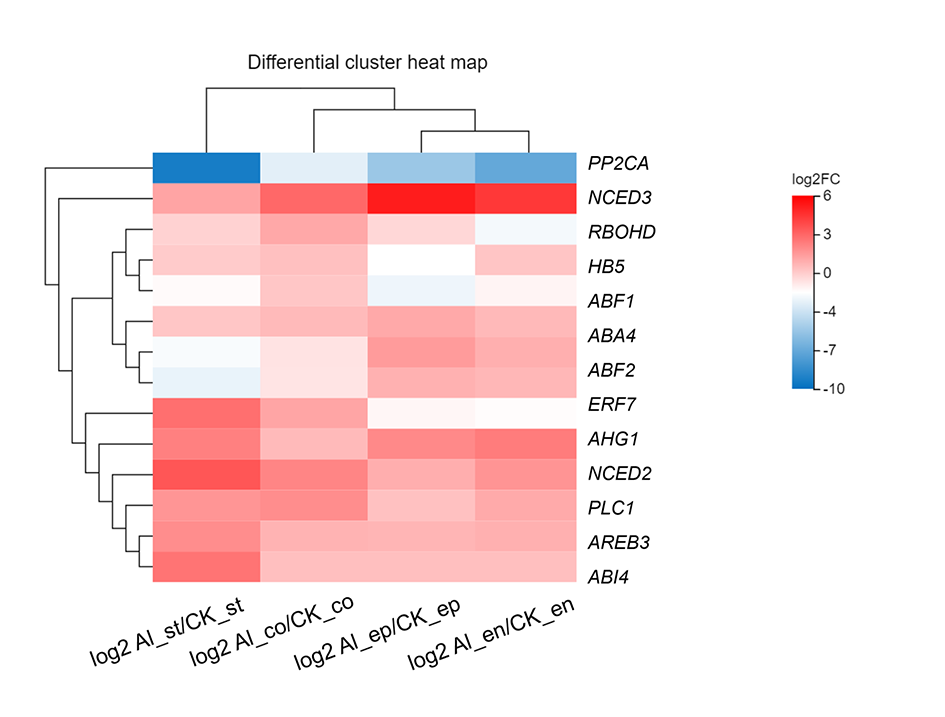

Supplement: Supplementary file 14 — FS14. [file PCE-48-7775-s018.tif]

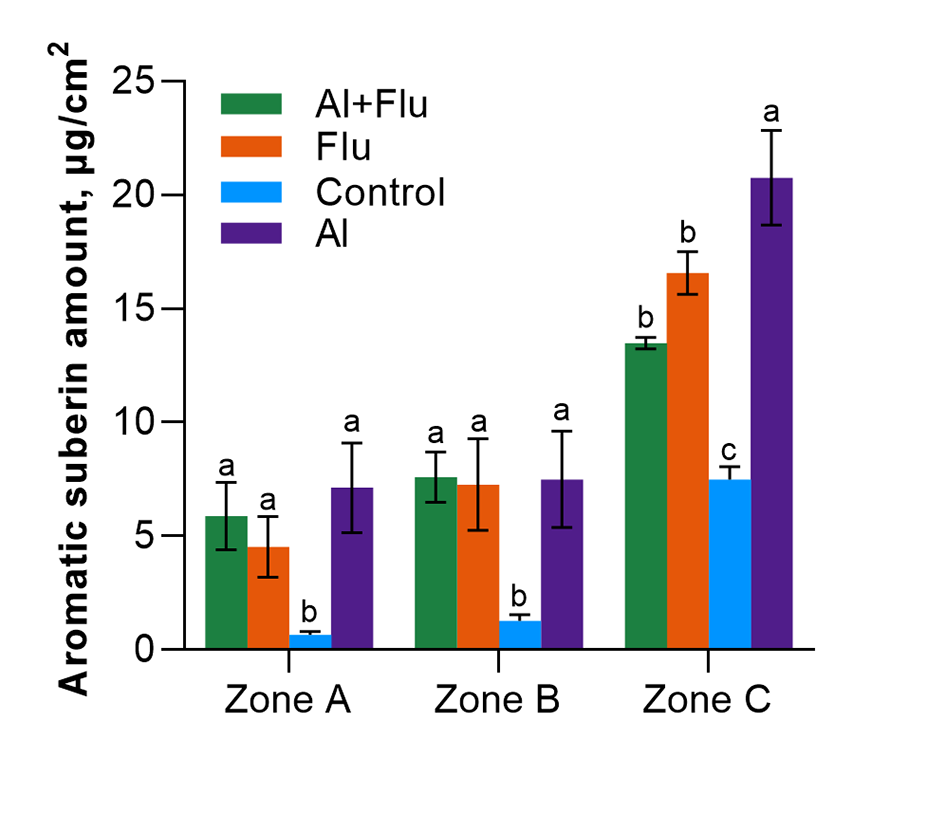

Supplement: Supplementary file 15 — FS15. [file PCE-48-7775-s022.tif]
