# Supplementary material for: Regulatory Programmes Driving Suberin Plasticity Under Aluminium Stress in Barley Roots
Source: Plant Cell Environ. 2025 Jul 17;48(11):7775–91. doi: 10.1111/pce.70075 (PMC12502035; doi:10.1111/pce.70075)
Supplement: Supplementary file 22 — supmat. [file PCE-48-7775-s008.docx]

**Supplementary data**

**Table S1.** Primers used in this study.

**Table S2.** Complete list of differentially expressed genes.

**Table S3.** DEGs and TPM values of barleys in response to Al stress**.**

**Table S4.** Endodermis specific genes.

**Table S5.** KEGG enrichment analysis of cutin suberin wax pathway in the barley endodermis in response to Al stress.

**Table S6.** WGCNA data.

**Fig. S1.** Total amounts of suberin in barley roots grown under different pH conditions.

**Fig. S2.** Amounts of substance classes of aliphatic suberin in barley seminal roots grown under different conditions.

**Fig. S3**. Tissue-specific expression of selected barley genes in root tissues under control conditions.

**Fig. S4.** Expressions of selected suberin-biosynthesis genes of barley under different conditions.

**Fig. S5.** Expression analysis of HvCYP86A1 and HvCYP86B1 genes of barley using qPCR.

**Fig. S6.** The correlation between Module Membership and Gene Significance.

**Fig. S7.** Protein sequence of *cyp86b1* mutants.

**Fig. S8.** Effect of Al on shoot and root dry weight of wild type and transgenic barley plants.

**Fig. S9.** Aromatic suberin amount in barley mutant.

**Fig. S10.** Amounts of monomers of aliphatic suberin in different zones of barley mutant roots.

**Fig. S11.** Amounts of monomers of aliphatic suberin in different zones of barley mutant roots under Al conditions.

**Fig. S12.** Amounts of substance classes of aliphatic suberin in wild type and transgenic barley seminal roots.

**Fig. S13.** Effect of Al stress on Flavonoid index, Anthocyanin index, Nitrogen Balance Index of GPF and barley mutants.

**Fig. S14.** Expressions of selected ABA pathway genes of barley under different conditions.

**Fig. S15.** Aromatic suberin amount in barley roots under Flu treatment.
